# Transforming family caregiving: professionalising unpaid care work for sustainable development

**DOI:** 10.5588/pha.23.0055

**Published:** 2024-03-01

**Authors:** B. N. Christian

**Affiliations:** Hospitals Management Board, Akwa Ibom State, Nigeria

**Keywords:** UN Sustainable Development Goals, family caregiving, unpaid care work, gender equity, economic empowerment

Nigeria is witnessing a remarkable demographic transformation, marked by a rapid increase in its elderly population.^1^ Projections indicate that there will be a significant increase in the number of citizens aged 65 and above in the coming years, from 9.4 million in 2020 to 25.5 million by 2050, a staggering 171% increase.^1^ This poses a major challenge as the demand for long-term care and the burden on informal family caregiving structures is predominantly borne by women.^2–4^ Caregivers face various challenges affecting different aspects of their lives, such as their work and education opportunities, social and personal relationships, financial security, and mental and physical well-being.^4^ Caregiving perpetuates existing gender roles in Nigerian society, limiting women’s choices, and is usually undertaken in unfavourable conditions with no information or training.^5^ This vital, but often unnoticed, work remains unrewarded, and is absent from strategic health policies.^5^ In Nigeria, unpaid care work has yet to be officially recognised and integrated into national plans and policies.^6^ Although there is yet no data on the economic value of unpaid care work in Nigeria, on a global scale, women play a significant role in positively influencing the health of roughly 5 billion individuals and contributing approximately US$3 trillion to the economy annually, nearly half of this substantial contribution stemming from unpaid caregiving responsibilities.^7^ The increasing ageing population in Nigeria will amplify the need for family caregiving and elderly care, intensifying caregiver burdens and widening gender disparities. This trend will also lead to reduced workforce engagement, healthcare hurdles and mental health challenges among caregivers, impacting economic productivity, straining relationships and disrupting societal cohesion. The WHO emphasises the need for societal restructuring across various sectors to address this global demographic shift, aligning with the 2030 sustainable development goals (SDGs).^3^

Interestingly, this demographic shift presents an opportunity for Nigeria to harness its untapped potential. Over 50% of the population comprises young, working-age individuals,^8^ a resource waiting to be optimised through strategic investments in education, healthcare and robust policies. According to the International Labour Organisation, investment in the care economy to achieve the UN Sustainable Development Goals would create approximately 475 million jobs by 2030.^9^ Formalising unpaid care work involves acknowledging the economic and social value of the work and integrating it into formal systems, policies and frameworks. Professionalising family caregiving, would enhance the family caregivers’ status and conditions of employment.^9^ This can be achieved by the following: 1) providing formal education, certifications and ongoing training to family caregivers; 2) developing standardised practices and ethical standards; 3) elevating their social status through fair compensation and career advancement opportunities; and 4) implementing supportive policies for their rights and safety, raising awareness about the importance of care work, and advocating for policies that respect caregivers’ contributions.

For example, although there are no monetary compensations for unpaid care workers in Ethiopia, the health system recognises care workers and provides ongoing mentorship and certificates as incentives.^5^ By including family caregiving in national plans and policies, Nigeria can mitigate the challenges posed by its ageing population, foster gender equality and propel economic empowerment. This would realise the triple gender dividend of better health, gender equity and economic growth.^7^ This transformation is not just about recognising the pivotal role of caregivers, it is an investment in a more equitable, prosperous future.

In conclusion, Nigeria has an opportunity to transform the caregiving sector. By harnessing the potential of the caregivers, the nation can close gender gaps, ensure health rights for older adults and create decent employment opportunities for its youth. Research on the economic value of unpaid care work is imperative for planning and action. It is time to formalise and professionalise care work to pave the way for a more inclusive and thriving society in Nigeria and other regions of the world.
